# Phosphonylated Acyclic Guanosine Analogues with the 1,2,3-Triazole Linker

**DOI:** 10.3390/molecules201018789

**Published:** 2015-10-16

**Authors:** Iwona E. Głowacka, Graciela Andrei, Dominique Schols, Robert Snoeck, Dorota G. Piotrowska

**Affiliations:** 1 Bioorganic Chemistry Laboratory, Faculty of Pharmacy, Medical University of Lodz, 90-151 Lodz, Muszyńskiego 1, Poland; E-Mail: dorota.piotrowska@umed.lodz.pl; 2 Rega Institute for Medical Research, KU Leuven, Minderbroedersstraat 10, B-3000 Leuven, Belgium; E-Mails: Graciela.Andrei@rega.kleuven.be (G.A.); Dominique.Schols@rega.kleuven.be (D.S.); Robert.Snoeck@rega.kleuven.be (R.S.)

**Keywords:** azidophosphonates, acyclonucleotides, 1,2,3-triazoles, cycloaddition, antiviral, cytostatic

## Abstract

A novel series of {4-[(2-amino-6-chloro-9*H*-purin-9-yl)methyl]-1*H*-1,2,3-triazol-1-yl}alkylphosphonates and {4-[(2-amino-6-oxo-1,6-dihydro-9*H*-purin-9-yl)methyl]-1*H*-1,2,3-triazol-1-yl}alkylphosphonates as acyclic analogues of guanosine were synthesized and assessed for antiviral activity against a broad range of DNA and RNA viruses and for their cytostatic activity toward three cancerous cell lines (HeLa, L1210 and CEM). They were devoid of antiviral activity; however, several phosphonates were found slightly cytostatic against HeLa cells at an IC_50_ in the 80–210 µM range. Compounds (1*R*,2*S*)-**17k** and (1*S*,2*S*)-**17k** showed the highest inhibitory effects (IC_50_ = 15–30 µM) against the proliferation of murine leukemia (L1210) and human T-lymphocyte (CEM) cell lines.

## 1. Introduction

**Figure 1:**
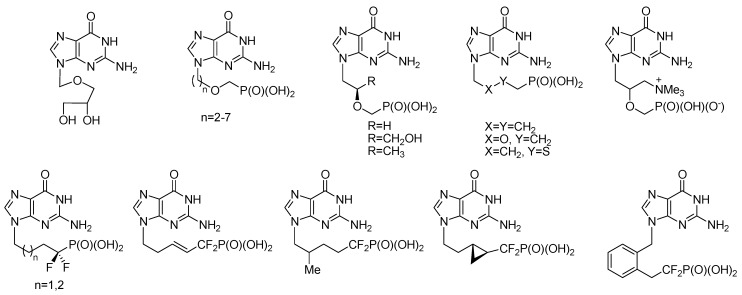
Known biologically-active acyclic analogues of guanosine.

An effective treatment for viral infections is one of the most difficult goals of contemporary medicine. The discovery of acyclic nucleosides/nucleotides, which act as antimetabolites, had a significant impact on the progress in the therapy of viral infections [1,2]. Among them, adefovir is active against DNA viruses and retroviruses [3,4,5], whereas tenofovir exhibits high potency and selectivity against HIV-1 and HIV-2 viruses and hepatitis B virus [6,7]. Ganciclovir [8,9,10] and its prodrug with improved oral bioavailability, valganciclovir [11,12], are used for the treatment of cytomegalovirus infections. Cidofovir [5,13,14] shows activity against herpes viruses, including cytomegalovirus, as well as adeno- and pox-viruses. The specificity of the antiviral activity of the compounds already known strongly depends on the structural features of the aliphatic chain installed as a sugar ring replacer, whereas a choice of nucleobases is mostly limited to adenine, guanine and 2,6-diaminopurine. Various guanine-containing analogues of nucleosides have been reported as potent antiviral agents (Figure 1) [15,16,17,18,19,20,21,22,23,24,25,26,27,28,29,30]. Moreover, acyclic analogues of nucleotides having guanine and hypoxanthine as a nucleobase with antimalarial activity have also been reported [26,31]. Phosphorylation of nucleosides and their structural analogues is inefficient, and at the same time, it appears to be one of the most important steps with implications on their activity, since the first step of phosphorylation is carried out by viral kinases. Therefore, several nucleotide analogues have been designed by incorporation of a phosphonate residue ((RO)_2_P(O)–CH_2_–) instead of a phosphate group ((RO)_2_P(O)–O–C(5′)) to avoid the first phosphorylation step and to ensure the stability of phosphonates to enzymatic hydrolysis [32,33,34,35].

**Figure 2:**
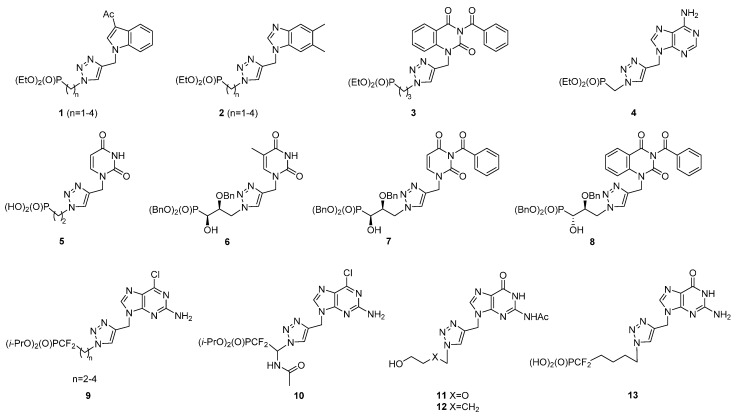
Examples of acyclic nucleotide analogues containing the 1,2,3-triazole linker.

In recent years, analogues of nucleotides containing various modifications of an acyclic fragment have been widely studied. Among them, extended linkers, including a 1,2,3-triazole moiety, were synthesized [36,37,38,39,40], and several compounds with promising anticancer (**1**–**3**) [38] and antiviral (**3**–**8**) [37,38,39] properties were found. Although various canonical nucleobases and their mimetics were applied, only a few acyclic guanosine, as well as 2-amino-6-chloropurine analogues containing the 1,2,3-triazole linker (**9**–**13**) have been obtained so far (Figure 2) [36,40,41,42,43]; however, among them, only Compound **13** was tested and revealed inhibitory activity against thymidine phosphorylase [40].

**Scheme 1:**
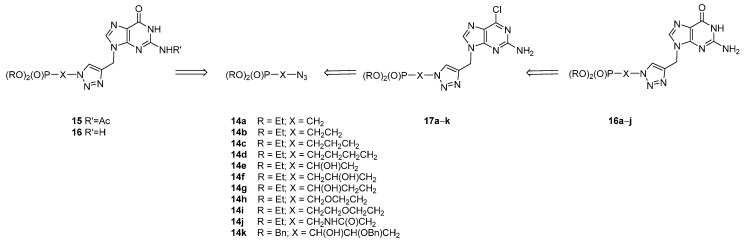
Retrosynthesis of acyclic phosphonate guanosine analogues.

As a continuation of our ongoing project directed towards biologically-active acyclic analogues of nucleotides with the 1,2,3-triazole linker, a new series of analogues **16** and **17** containing 2-amino-6-chloropurine and guanine as nucleobases has been designed to study their antiviral and cytostatic properties. To install a guanine moiety at C4 in the 1,2,3-triazole ring, two strategies were used. The dipolar cycloaddition of the respective azidoalkylphosphonates **14** to propargylated guanines should directly lead to Compound **15** or **16** [41,42,43], whereas application of 2-amino-6-chloro-9-propargylpurine as dipolarophile in the reaction with azides **14** should produce Compound **17** to be subsequently transformed into **16** [40,44,45,46,47] (Scheme 1).

Generally, in order to secure sufficient bioavailability of active phosphonate nucleotide analogues, they are administered as prodrugs, namely the respective phosphonate esters or amides [33,34,35,48,49,50,51,52,53]. For this reason, we designed guanosine analogues **16** and **17** as the respective phosphonate esters to ensure sufficient membrane permeability. Moreover, our recent experiences clearly supported the strategy to prepare diesters rather than free phosphonic acids, which are completely ionized at physiological pH. Indeed, we found several examples of active diesters in the class of 1,2,3-triazole phosphonates, whereas the respective free acids appeared inactive [38,39,54].

## 2. Results and Discussion

### 2.1. Chemistry

Propargylated 2-amino-6-chloropurine **19** [29,36,40,45,55], guanine **18a** [29,44,45,46,47,55] and *N*^2^-acetylguanine **18b** [42,43,56], as well as all azidoalkylphosphonates **14a**–**k** [37,38,57,58,59,60,61,62] are known compounds and were obtained according to the literature procedures.

**Scheme 2:**
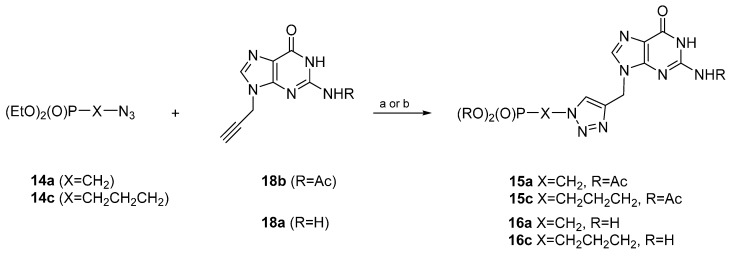
Attempts at synthesizing acyclic guanosine analogues from guanines **18a** and **18b**. *Reaction and conditions*: (a) Toluene, reflux, 72 h; (b) CuSO_4_ × 5H_2_O, sodium ascorbate, EtOH–H_2_O, 35–40 °C, 3 h, microwave (MW).

Although cycloadditions of propargylated guanines **18a** (R = H) and **18b** (R = Ac) to various azides have previously been mentioned [41,42,43], in our hands, the reaction of **18a**, as well as **18b** with the azidomethylphosphonate **14a** failed because of the low solubility of guanines **18a** and **18b** (Scheme 2). Attempts at running a cycloaddition of the phosphonate **14a** with propargylguanine **18a** at 110 °C in toluene resulted in the recovery of starting materials only. Similarly, when the 3-azidopropylphosphonate **14c** was treated with **18a** or **18b** at 110 °C in toluene, as well as under microwave (MW) irradiation in aqueous ethanol, no traces of cycloadducts were observed.

**Scheme 3:**
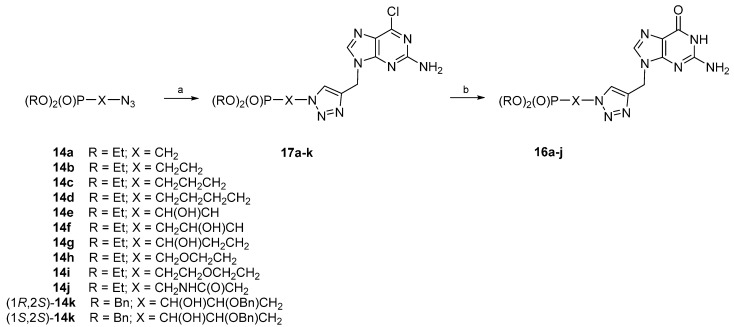
Synthesis of Compounds **17a**‒**k** and **16a**‒**j**. *Reaction and conditions*: (a) 2-amino-6-chloro-9-propargylpurine **19**, CuSO_4_ × 5H_2_O, sodium ascorbate, EtOH–H_2_O, 35–40 °C, 15 min, microwave (MW); (b) 75% TFA, 24 h, r.t.

For this reason, we turned to 2-amino-6-chloro-9-propargylpurine **19**, which was found to be sufficiently soluble in the reaction medium and reacted with the respective ω-azidoalkylphosphonates **14** to form the intermediate 2-amino-6-chloropurines **17**, which were transformed into guanine analogues in the last step. Thus, azides **14** were subjected to cycloaddition with Compound **19** in the presence of Cu(I) salt under microwave irradiation to give 1,2,3-triazoles **17a**–**k**. Reactions were complete at 35–40 °C within 15 min (Scheme 3). Subsequently, **17a**–**k** were treated with 75% trifluoroacetic acid to provide acyclic guanosine analogues **16a**–**k** in good yields (92%–98%). However, attempts at preparing (1*R*,2*S*)-**16k** and (1*S*,2*S*)-**16k** failed, since the treatment of (1*R*,2*S*)-**17k** and (1*S*,2*S*)-**17k** with trifluoroacetic acid led to severe decomposition. All final compounds were purified by chromatography, and solids were finally recrystallized; their purity was ascertained by NMR spectroscopic methods and elemental analysis.

### 2.2. Antiviral Activity and Cytostatic/Cytotoxic Evaluation

All phosphonates **16a**–**j** and **17a**–**k** were evaluated for their antiviral activities against a wide variety of DNA and RNA viruses using the following cell-based assays: (1) human embryonic lung (HEL) cell cultures: herpes simplex virus-1 (KOS), herpes simplex virus-2 (G), vaccinia virus, vesicular stomatitis virus, herpes simplex virus-1 (TK^−^ KOS ACV^r^) and adenovirus-2, cytomegalovirus (AD-169 strain and Davis strain) and varicella-zoster virus (TK^+^ VZV stain and TK^−^ VZV stain); (2) HeLa cell cultures: vesicular stomatitis virus, Coxsackie virus B4 and respiratory syncytial virus; (3) Vero cell cultures: para-influenza-3 virus, reovirus-1, Sindbis virus, Coxsackie virus B4, Punta Toro virus; (4) Crandell-Rees feline kidney (CRFK) cell cultures: feline corona virus (FIPV) and feline herpesvirus (FHV); and (5) Madin-Darby canine kidney (MDCK) cell cultures: influenza A virus H1N1 subtype, influenza A virus H3N2 subtype and influenza B virus. Ganciclovir, cidofovir, acyclovir, brivudine, (*S*)-9-(2,3-dihydroxypropyl)adenine ((*S*)-DHPA), *Hippeastrum* hybrid agglutinin (HHA), *Urtica dioica* agglutinin (UDA), dextran sulfate (molecular weight 5000, DS-5000), ribavirin, oseltamivir carboxylate, amantadine and rimantadine were used as the reference compounds. The antiviral activity was expressed as the EC_50_: the compound concentration required to reduce virus-induced cytopathogenicity by 50%. Unfortunately, no inhibitory activity against any virus was detected for the evaluated compounds at 250 µM.

The cytotoxicity of the tested compounds toward the uninfected host cells was defined as the minimum cytotoxic concentration (MCC) that causes a microscopically-detectable alteration of normal cell morphology. The 50% cytotoxic concentration (CC_50_), causing a 50% decrease in cell viability, was determined using a colorimetric 3-(4,5-dimethylthiazol-2-yl)-5-(3-carboxymethoxyphenyl)-2-(4-sulfophenyl)-2*H*-tetrazolium (MTS) assay system. The cytostatic activity of the tested compounds was defined as the 50% cytostatic inhibitory concentration (IC_50_), causing a 50% decrease in cell proliferation, and was determined against murine leukemia L1210, human T-lymphocyte CEM and human cervix carcinoma HeLa cells (Table 1).

**Table 1:** Inhibitory effect of the tested compounds against the proliferation of murine leukemia (L1210), human T-lymphocyte (CEM) and human cervix carcinoma cells (HeLa).

Compounds 16a–k and 17a–k	IC_50_^a^ (µM)		
	L1210	CEM	HeLa
**17a**	283 ± 17	≥250	≥250
**17b**	≥250	>250	227 ± 32
**17c**	227 ± 32	>250	≥250
**17d**	>250	>250	≥250
**17e**	>250	>250	>250
**17f**	>250	>250	>250
**17g**	>250	>250	>250
**17h**	>250	≥250	>250
**17i**	>250	>250	>250
**17j**	>250	>250	>250
(1*R*,2*S*)-**17k**	21 ± 2	26 ± 8	90 ± 33
(1*S*,2*S*)-**17k**	16 ± 6	30 ± 16	84 ± 12
**16a**	>250	>250	>250
**16b**	>250	>250	>250
**16c**	>250	>250	138 ± 52
**16d**	>250	>250	148 ± 25
**16e**	>250	>250	206 ± 49
**16f**	>250	>250	195 ± 78
**16g**	>250	>250	185 ± 35
**16h**	>250	>250	≥250
**16i**	>250	>250	210 ± 13
**16j**	>250	>250	212 ± 54
5-fluorouracil	0.33 ± 0.17	18 ± 5	0.54 ± 0.12

^a^ 50% Inhibitory concentration or compound concentration required to inhibit tumor cell proliferation by 50%.

None of the tested compounds affected cell morphology of HEL, HeLa, Vero, MDCK and CRFK cells at concentrations up to 100 μM. Instead, several compounds appeared slightly cytostatic, selectively against HeLa cells at an IC_50_ in the 80–210 µM range. From the entire library of compounds, only (1*R*,2*S*)-**17k** and (1*S*,2*S*)-**17k** appeared to be the most active toward all tested cancerous cell lines, and they showed the highest inhibitory effect (IC_50_ = 16–30 µM) against the proliferation of murine leukemia (L1210) and human T-lymphocytes (CEM). 

The significantly higher activity of dibenzyl phosphonates (1*R*,2*S*)-**17k** and (1*S*,2*S*)-**17k** is probably due to their better penetration through cell membranes when compared to the other compounds in the series **17**, as well as **16,** which all were tested as diethyl esters.

## 3. Experimental Section

### 3.1. General

^1^H-NMR were taken in CDCl_3_ or CD_3_OD on the following spectrometers: Varian Mercury-300 (Varian NMR Instrument, Palo Alto, CA, USA) with TMS as an internal standard; chemical shifts δ in ppm with respect to TMS; coupling constants *J* in Hz. ^13^C-NMR spectra were recorded on Varian Mercury-300 (Varian NMR Instrument, Palo Alto, CA, USA) and Bruker Avance III spectrometers (Bruker Instruments, Karlsruhe, Germany) at 75.5 and 151 MHz, respectively. ^31^P-NMR spectra were taken in CDCl_3_ or CD_3_OD on a Varian Mercury-300 (Varian NMR Instrument, Palo Alto, CA, USA) at 121.5 MHz.

IR spectral data were measured on an Infinity MI-60 FT-IR spectrometer (ATI Instruments North America—Mattson, Madison, WI, USA). Melting points were determined on a Boetius apparatus (VEB Kombinat NAGEMA, Dresden, DDR—Currently Germany) and are uncorrected. Elemental analyses were performed by the Microanalytical Laboratory of this faculty on a Perkin Elmer PE 2400 CHNS analyzer (Perkin-Elmer Corp., Norwalk, CT, USA).

The following adsorbents were used: column chromatography, Merck silica gel 60 (70–230 mesh); analytical TLC, Merck TLC plastic sheets silica gel 60 F_254_. TLC plates were developed in chloroform−methanol solvent systems. Visualization of spots was effected with iodine vapors. All solvents were purified by methods described in the literature.

Microwave irradiation experiments were carried out in 50-mL glass vials in a microwave reactor Plazmatronika RM 800 (Plazmatronika, Wrocław, Poland).

### 3.2. General Procedure for the Synthesis of ***17a**–**k***

To a solution of the respective azidoalkylphosphonate **14** (1.00 mmol) in EtOH (1 mL) and H_2_O (1 mL), CuSO_4_ × 5H_2_O (0.05 mmol), sodium ascorbate (0.10 mmol) and 2-amino-6-chloro-9-propargyl-purine (1.00 mmol) were added. The suspension was irradiated in the microwave reactor (Plazmatronika RM 800, 800 W, Plazmatronika, Wrocław, Poland) at 35–40 °C for 15 min. Solvents were removed by vacuum evaporation, and the residue was suspended in chloroform (5 mL) and filtered through a layer of Celite. The solution was concentrated *in vacuo*, and the crude product was purified on a silica gel column with chloroform–methanol mixtures (50:1, 20:1 or 10:1, *v*/*v*) to give the appropriate 1,2,3-triazoles **17**.

*Diethyl {4-[(2-amino-6-chloro-9H-purin-9-yl)methyl]-1H-1,2,3-triazol-1-yl}methylphosphonate* (**17a**): A white solid (after crystallization from ethyl acetate), yield 88%; m.p. = 138–139 °C; IR (KBr): ν = 3327, 3211, 2988, 1796, 1613, 1565, 1518, 1468, 1408, 1241 cm^−1^; ^1^H-NMR (300 MHz, CDCl_3_): δ = 7.94 (d, *J* = 0.8 Hz, 1H, HC5′), 7.93 (s, 1H), 5.38 (s, 2H, CH_2_), 5.31 (s, 2H, NH_2_), 4.76 (d, *J* = 13.3 Hz, 2H, PCH_2_), 4.15–4.04 (m, 4H, 2 × POC*H*_2_CH_3_), 1.26 (t, *J* = 6.9 Hz, 3H, POCH_2_C*H*_3_); 1.25 (t, *J* = 6.9 Hz, 3H, POCH_2_C*H*_3_); ^13^C-NMR (75.5 MHz, CDCl_3_): δ = 159.43, 153.44, 150.97, 142.27, 142.18, 124.63, 124.52 (d, *J* = 5.2 Hz, C5′), 63.81 (d, *J* = 6.6 Hz, POC), 46.08 (d, *J* = 155.2 Hz, PC), 38.70, 16.46 (d, *J* = 5.5 Hz, POC*C*), 16.42 (d, *J* = 5.5 Hz, POC*C*); ^31^P-NMR (121.5 MHz, CDCl_3_): δ = 15.93 ppm. Anal. calcd. for C_13_H_18_ClN_8_O_3_P: C, 38.96; H, 4.53; N, 27.96. Found: C, 38.97; H, 4.33; N, 27.72.

*Diethyl 2-{4-[(2-amino-6-chloro-9H-purin-9-yl)methyl]-1H-1,2,3-triazol-1-yl}ethylphosphonate* (**17b**): A white solid (after crystallization from ethyl acetate), yield 91%; m.p. = 145–146 °C; IR (KBr): ν = 3325, 3211, 2985, 1796, 1613, 1565, 1518, 1468, 1408, 1223, 1050 cm^−1^; ^1^H-NMR (300 MHz, CDCl_3_): δ = 7.94 (s, 1H, HC5′), 7.76 (s, 1H), 5.37 (s, 2H, CH_2_), 5.21 (s, 2H, NH_2_), 4.62 (dt, *J* = 13.4 Hz, *J* = 7.4 Hz, 2H, PCH_2_C*H*_2_), 4.10–4.00 (m, 4H, 2 × POC*H*_2_CH_3_), 2.40 (dt, *J* = 18.3 Hz, *J* = 7.4 Hz, 2H, PC*H*_2_), 1.26 (t, *J* = 7.1 Hz, 6H, POCH_2_C*H*_3_); ^13^C-NMR (75.5 MHz, CDCl_3_): δ = 159.36, 153.34, 150.93, 142.19, 142.13, 141.81, 124.58, 123.73 (d, *J* = 4.0 Hz, C5′), 62.34 (d, *J* = 6.6 Hz, POC), 44.74 (PC*C*), 38.55, 26.99 (d, *J* = 141.4 Hz, PC), 16.44 (d, *J* = 5.7 Hz, POC*C*), 16.40 (d, *J* = 5.7 Hz, POC*C*); ^31^P-NMR (121.5 MHz, CDCl_3_): δ = 25.81 ppm. Anal. calcd. for C_14_H_20_ClN_8_O_3_P: C, 40.54; H, 4.86; N, 27.01. Found: C, 40.63; H, 4.60; N, 27.04.

*Diethyl 3-{4-[(2-amino-6-chloro-9H-purin-9-yl)methyl]-1H-1,2,3-triazol-1-yl}propylphosphonate* (**17c**): White powder, yield 86%; m.p. = 74–76 °C; IR (KBr): ν = 3390, 3210, 2985, 1797, 1617, 1564, 1468, 1409, 1215, 1025 cm^−1^; ^1^H-NMR (300 MHz, CDCl_3_): δ = 7.94 (s, 1H, HC5′), 7.72 (s, 1H), 5.37 (s, 2H, CH_2_), 5.28 (s, 2H, NH_2_), 4.46 (t, *J* = 6.9 Hz, 2H, PCH_2_CH_2_C*H*_2_), 4.14–4.02 (m, 4H, 2 × POC*H*_2_CH_3_), 2.21 (dqu, *J* = 18.7 Hz, *J* = 6.9 Hz, 2H, PCH_2_C*H*_2_), 1.69 (dt, *J* = 18.7 Hz, *J* = 7.9 Hz, 2H, PC*H*_2_), 1.31 (t, *J* = 7.2 Hz, 6H, POCH_2_C*H*_3_); ^13^C-NMR (75.5 MHz, CDCl_3_): δ = 159.34, 153.42, 151.09, 142.24, 142.18, 141.93, 124.79, 123.49 (d, *J* = 3.1 Hz, C5′), 62.07 (d, *J* = 6.6 Hz, POC), 50.16 (d, *J* = 14.6 Hz, PCC*C*), 38.69, 23.67 (d, *J* = 4.6 Hz, PC*C*), 22.46 (d, *J* = 142.9 Hz, PC), 16.62 (d, *J* = 5.7 Hz, POC*C*), 16.56 (d, *J* = 5.7 Hz, POC*C*); ^31^P-NMR (121.5 MHz, CDCl_3_): δ = 30.26 ppm. Anal. calcd. for C_15_H_22_ClN_8_O_3_P: C, 42.01; H, 5.17; N, 26.13. Found: C, 41.92; H, 5.14; N, 25.83.

*Diethyl 4-{4-[(2-amino-6-chloro-9H-purin-9-yl)methyl]-1H-1,2,3-triazol-1-yl}butylphosphonate* (**17d**): Colorless oil, yield 93%; IR (film): ν = 3324, 3210, 2983, 1613, 1562, 1467, 1408, 1215, 1025 cm^−1^; ^1^H-NMR (300 MHz, CDCl_3_): δ = 7.94 (s, 1H, HC5′), 7.65 (s, 1H), 5.37 (s, 2H, CH_2_), 5.40–5.00 (very br s, 2H, NH_2_), 4.36 (t, *J* = 6.9 Hz, 2H, PCCCC*H*_2_), 4.20–4.00 (m, 4H, 2 × POC*H*_2_CH_3_), 2.02 (qu, *J* = 6.9 Hz, 2H, PCCC*H*_2_), 1.80–1.60 (m, 4H, PCC*H*_2_ and PC*H*_2_), 1.30 (t, *J* = 7.0 Hz, 6H, 2 × POCH_2_C*H*_3_); ^13^C-NMR (151 MHz, CDCl_3_): δ = 159.47, 153.50, 151.01, 142.26, 141.95, 124.67, 123.06, 61.69 (d, *J* = 6.6 Hz, 2 × POC), 49.76, 38.54, 30.43 (d, *J* = 15.0 Hz, PCC*C*), 24.66 (d, *J* = 141.9 Hz, PC), 19.48 (d, *J* = 4.9 Hz, PC*C*), 16.30 (d, *J* = 6.0 Hz, POC*C*); ^31^P-NMR (121.5 MHz, CDCl_3_): δ = 31.33 ppm. Anal. calcd. for C_16_H_24_ClN_8_O_3_P: C, 43.40; H, 5.46; N, 25.30. Found: C, 43.21; H, 5.40; N, 25.23.

*Diethyl 2-{4-[(2-amino-6-chloro-9H-purin-9-yl)methyl]-1H-1,2,3-triazol-1-yl}-1-hydroxyethylphosphonate* (**17e**): White solid (after column chromatography and crystallization from ethyl acetate), yield 81%; m.p. = 174–175 °C; IR (KBr): ν = 3433, 3218, 2986, 1618, 1565, 1468, 1409, 1215, 1021 cm^−1^; ^1^H-NMR (300 MHz, CDCl_3_): δ = 7.86 (s, 1H, HC5′), 7.83 (s, 1H), 5.40 (br t, *J* = 4.6 Hz, 1H, OH), 5.73 (s, 2H, NH_2_), 5.21 (AB, *J*_AB_ = 15.5 Hz, 1H, *H*CH), 5.20 (AB, *J*_AB_ = 15.5 Hz, 1H, HC*H*), 4.85–4.76 (m, 1H), 4.51–4.38 (m, 2H), 4.27–4.14 (m, 4H, 2 × POC*H*_2_CH_3_), 1.35 (t, *J* = 7.0 Hz, 3H, POCH_2_C*H*_3_), 1.33 (t, *J* = 7.0 Hz, 3H, POCH_2_C*H*_3_); ^13^C-NMR (75.5 MHz, CDCl_3_): δ = 159.50, 153.13, 150.61, 142.43, 141.49, 125.41, 123.93, 66.75 (d, *J* = 167.2 Hz, PC), 63.64 (d, *J* = 7.2 Hz, POC), 63.56 (d, *J* = 7.2 Hz, POC), 52.02 (PC*C*), 38.56, 16.63 (d, *J* = 5.7 Hz, 2 × POC*C*); ^31^P-NMR (121.5 MHz, CDCl_3_): δ = 20.67 ppm. Anal. calcd. for C_14_H_20_ClN_8_O_4_P: C, 39.03; H, 4.68; N, 26.01. Found: C, 39.25; H, 4.42; N, 26.05.

*Diethyl 3-{4-[(2-amino-6-chloro-9H-purin-9-yl)methyl]-1H-1,2,3-triazol-1-yl}-2-hydroxypropylphosphonate* (**17f**): Colorless oil, yield 89%; IR (film): ν = 3346, 3218, 3218, 2985, 1618, 1564, 1468, 1409, 1220, 1025 cm^−1^; ^1^H-NMR (300 MHz, CDCl_3_): δ = 7.93 (s, 1H, HC5′), 7.87 (s, 1H), 5.30 (s, 4H, CH_2_ and NH_2_), 4.60–4.47 (m, 1H, PCCC*H*_a_H_b_), 4.45–4.30 (m, 1H, PCCCH_a_*H*_b_), 4.20–4.00 (m, 4H, 2 × POC*H*_2_CH_3_), 2.10–1.70 (m, 4H, OH, PCH_2_), 1.33 (t, *J* = 7.1 Hz, 3H, POCH_2_C*H*_3_), 1.31 (t, *J* = 7.1 Hz, 3H, POCH_2_C*H*_3_); ^13^C-NMR (75.5 MHz, CDCl_3_): δ = 159.44, 153.33, 150.71, 142.46, 141.58, 125.14, 124.27, 65.46 (PC*C*), 62.50 (d, *J* = 6.3 Hz, POC), 62.32 (d, *J* = 6.6 Hz, POC), 56.21 (d, *J* = 15.2 Hz, PCC*C*), 38.65, 31.09 (d, *J* = 139.7 Hz, PC), 16.53 (d, *J* = 6.8 Hz, 2 × POC*C*); ^31^P-NMR (121.5 MHz, CDCl_3_): δ = 28.62 ppm. Anal. calcd. for C_15_H_22_ClN_8_O_4_P: C, 40.50; H, 4.99; N, 25.19. Found: C, 40.55; H, 5.06; N, 25.30.

*Diethyl 3-{4-[(2-amino-6-chloro-9H-purin-9-yl)methyl]-1H-1,2,3-triazol-1-yl}-1-hydroxypropylphosphonate* (**17g**): White powder, yield 95%; m.p. = 124–126 °C; IR (KBr): ν = 3405, 3217, 2983, 1618, 1563, 1468, 1409, 1217, 1022 cm^−1^; ^1^H-NMR (300 MHz, CDCl_3_): δ = 8.01 (s, 1H, HC5′), 7.75 (s, 1H), 5.38 (s, 2H, CH_2_), 4.58 (dd, *J* = 7.5 Hz, *J* = 5.8 Hz, 2H, PCCC*H*_2_), 4.21–4.08 (m, 4H, 2 × POC*H*_2_CH_3_), 3.77 (ddd, *J* = 12.4 Hz, *J* = 6.2 Hz, *J* = 3.2 Hz, 1H, PC*H*), 2.44–2.14 (m, 4H, PCCH_2_ and NH_2_), 1.32 (t, *J* = 7.1 Hz, 3H, POCH_2_C*H*_3_), 1.29 (t, *J* = 7.1 Hz, 3H, POCH_2_C*H*_3_); ^13^C-NMR (151 MHz, CDCl_3_): δ = 160.33, 153.68, 150.22, 142.80, 142.24, 124.02, 123.42, 63.67 (d, *J* = 168.2 Hz, PC), 63.00 (d, *J* = 7.0 Hz, POC), 62.65 (d, *J* = 7.0 Hz, POC), 46.30 (d, *J* = 15.6 Hz, PCC*C*), 38.05, 31.70 (d, *J* = 4.0 Hz, PC*C*) 15.40 (d, *J* = 5.5 Hz, POC*C*), 15.30 (d, *J* = 5.5 Hz, POC*C*); ^31^P-NMR (121.5 MHz, CDCl_3_): δ = 24.00 ppm. Anal. calcd. for C_15_H_22_ClN_8_O_4_P: C, 40.50; H, 4.99; N, 25.19. Found: C, 40.54; H, 4.79; N, 24.97.

*Diethyl 2-{4-[(2-amino-6-chloro-9H-purin-9-yl)methyl]-1H-1,2,3-triazol-1-yl}-ethoxymethylphosphonate* (**17h**): Colorless oil, yield 88%; IR (film): ν = 2988, 1798, 1633, 1614, 1470, 1410, 1220, 1025 cm^−1^; ^1^H-NMR (300 MHz, CDCl_3_): δ = 8.02 (s, 1H, HC5′), 7.96 (s, 1H), 5.37 (s, 2H, CH_2_), 4.56 (t, *J* = 4.8 Hz, 2H, PCH_2_OCH_2_C*H*_2_), 4.18–4.08 (m, 4H, 2 × POC*H*_2_CH_3_), 3.95 (t, *J* = 4.8 Hz, 2H, PCOC*H*_2_CH_2_), 3.77 (d, *J* = 8.7 Hz, 2H, PCH_2_O), 2.00 (br s, 2H, NH_2_), 1.31 (t, *J* = 7.2 Hz, 6H, 2 × POCH_2_C*H*_3_); ^13^C-NMR (151 MHz, CDCl_3_): δ = 159.53, 153.55, 151.00, 142.22, 141.87, 124.69, 124.44, 71.10 (d, *J* = 11.7 Hz, PCO*C*), 65.18 (d, *J* = 168.2 Hz, PC), 62.61 (d, *J* = 6.6 Hz, PO*C*), 50.10, 38.58, 16.37 (d, *J* = 5.5 Hz, POC*C*); ^31^P-NMR (121.5 MHz, CDCl_3_): δ = 20.60 ppm. Anal. calcd. for C_15_H_22_ClN_8_O_4_P: C, 40.50; H, 4.99; N, 25.19. Found: C, 40.37; H, 4.80; N, 25.10.

*Diethyl 2-{4-[(2-amino-6-chloro-9H-purin-9-yl)methyl]-1H-1,2,3-triazol-1-yl}-ethoxyethylphosphonate* (**17i**): Colorless oil, yield 95%; IR (film): ν = 3325, 3209, 2988, 1614, 1563, 1467, 1408, 1221, 1052, 1025 cm^−1^; ^1^H-NMR (300 MHz, CDCl_3_): δ = 7.99 (s, 1H, HC5′), 7.98 (s, 1H), 5.37 (s, 2H, CH_2_), 4.53 (dd, *J* = 5.0 Hz, *J* = 4.8 Hz, 2H, PCH_2_CH_2_OCH_2_C*H*_2_), 4.14–4.03 (m, 4H, 2 × POC*H*_2_CH_3_), 3.80 (dd, *J* = 5.3 Hz, *J* = 5.0 Hz, 2H, PCCOC*H*_2_CH_2_), 3.70 (dt, *J* = 15.3 Hz, *J* = 7.0 Hz, 2H, PCH_2_C*H*_2_O), 2.05 (dt, *J* = 18.5 Hz, *J* = 7.0 Hz, 2H, PC*H*_2_CH_2_O), 1.30 (t, *J* = 7.0 Hz, 6H, 2 × POCH_2_C*H*_3_); ^13^C-NMR (151 MHz, CDCl_3_): δ = 159.50, 153.55, 151.02, 142.28, 141.84, 124.73, 124.42, 68.82, 65.10 (d, *J* = 2.9 Hz, PC*C*O), 61.76 (d, *J* = 6.5 Hz, PO*C*), 50.23, 38.53, 26.66 (d, *J* = 141.0 Hz, PC), 16.30 (d, *J* = 6.2 Hz, POC*C*); ^31^P-NMR (121.5 MHz, CDCl_3_): δ = 28.55 ppm. Anal. calcd. for C_16_H_24_ClN_8_O_4_P: C, 41.88; H, 5.27; N, 24.42. Found: C, 42.01; H, 5.33; N, 24.60.

*Diethyl 2-{4-[(2-amino-6-chloro-9H-purin-9-yl)methyl]-1H-1,2,3-triazol-1-yl}-acetamidoethylphosphonate* (**17j**): White powder, yield 78%; m.p. = 193–195 °C; IR (KBr): ν = 3330, 3216, 2986, 1690, 1615, 1563, 1468, 1409, 1215, 1023 cm^−1^; ^1^H-NMR (300 MHz, CDCl_3_): δ = 7.96 (s, 1H, HC5′), 7.86 (s, 1H), 7.20 (br s, 1H, NHCO), 5.39 (s, 2H, CH_2_), 5.01 (s, 2H), 4.15–4.06 (m, 4H, 2 × POC*H*_2_CH_3_), 3.72 (dd, *J* = 11.9 Hz, *J* = 5.7 Hz, 2H, PC*H*_2_NH), 1.71 (br s, 2H, NH_2_), 1.29 (t, *J* = 6.9 Hz, 6H, 2 × POCH_2_C*H*_3_); ^13^C-NMR (151 MHz, DMSO-*d*_6_): δ = 165.91, 160.39, 154.37, 149.91, 143.43, 142.48, 125.41, 123.69, 62.32 (d, d, *J* = 6.1 Hz, PO*C*), 51.92, 38.65, 34.70 (d, *J* = 155.2 Hz, PC), 16.66 (d, *J* = 5.5 Hz, POC*C*); ^31^P-NMR (121.5 MHz, CDCl_3_): δ = 21.92 ppm. Anal. calcd. for C_15_H_21_ClN_9_O_4_P: C, 39.35; H, 4.62; N, 27.54. Found: C, 39.57; H, 4.51; N, 27.47.

*(1R,2S)-Dibenzyl 3-{4-[(2-amino-6-chloro-9H-purin-9-yl)methyl]-1H-1,2,3-triazol-1-yl}-2-benzyloxy-1-hydroxypropylphosphonate* ((1*R*,2*S*)*-***17k**): Colorless oil, yield 84%; ${\text{[α]}}_{D}^{20}$ = +9.6 (c = 1.12 in CHCl_3_); IR (film): ν = 3333, 3212, 1690, 1616, 1564, 1457, 1409, 1215, 1135 cm^−1^; ^1^H-NMR (300 MHz, CDCl_3_): δ = 7.96 (s, 1H, HC5′), 7.61 (s, 1H), 7.31–7.23 (m, 11H), 7.22–7.15 (m, 2H), 7.08–7.02 (m, 2H), 5.27 (AB, *J*_AB_ = 15.5 Hz, 1H, *H*CH), 5.22 (AB, *J*_AB_ = 15.5 Hz, 1H, HC*H*), 5.08–4.96 (m, 4H, 2 × POC*H*_2_Ph), 4.59 (dd, *J* = 14.1 Hz, *J* = 5.7 Hz, 1H, *H*CHN), 4.57 (d, *J* = 10.8 Hz, 1H, *H*CH-Ph), 4.47 (dd, *J* = 14.1 Hz, *J* = 7.0 Hz, 1H, HC*H*N), 4.32–4.20 (m, 1H, PCC*H*), 4.17 (d, *J* = 10.8 Hz, 1H, HC*H*-Ph), 3.91 (dd, *J* = 11.6 Hz, *J* = 3.2 Hz, 1H, PC*H*), 1.90–1.10 (very br s, 3H, NH_2_ and OH); ^13^C-NMR (151 MHz, CDCl_3_): δ = 159.18, 153.37, 151.45, 142.19, 141.81, 136.69, 135.77 (d, 5.8 Hz), 135.63 (d, 5.8 Hz), 128.77, 128.71, 128.69, 128.43, 128.22, 128.20, 128.17, 125.02, 124.53, 74.06, 68.71 (d, *J* = 7.4 Hz, PO*C*), 68.41 (d, *J* = 7.4 Hz, PO*C*), 68.28 (d, *J* = 162.0 Hz, PC), 50.66 (d, *J* = 11.4 Hz, PCC*C*), 38.52; ^31^P-NMR (121.5 MHz, CDCl_3_): δ = 22.35 ppm. Anal. calcd. for C_32_H_32_ClN_8_O_5_P: C, 56.93; H, 4.78; N, 16.60. Found: C, 56.80; H, 4.52; N, 16.44.

*(1S,2S)-Dibenzyl 3-{4-[(2-amino-6-chloro-9H-purin-9-yl)methyl]-1H-1,2,3-triazol-1-yl}-2-benzyloxy-1-hydroxypropylphosphonate* ((1*S*,2*S*)*-***17k**): White solid, yield 84%; ${\text{[α]}}_{D}^{20}$ = +22.9 (c = 0.096 in CHCl_3_); m.p. = 76–78 °C; IR (KBr): ν = 3384, 3213, 1617, 1563, 1457, 1409, 1214 cm^−1^; ^1^H-NMR (300 MHz, CDCl_3_): δ = 7.94 (s, 1H, HC5′), 7.64 (s, 1H), 7.38–7.20 (m, 11H), 7.20–7.10 (m, 2H), 7.00–6.95 (m, 2H), 5.26 (s, 2H, CH_2_), 5.04 (d, *J* = 8.5 Hz, 2H, POC*H*_2_Ph), 5.01 (d, *J* = 9.5 Hz, 2H, POC*H*_2_Ph),4.73 (dd, *J* = 14.5 Hz, *J* = 3.2 Hz, 1H, *H*CHN), 4.60 (dd, *J* = 14.6 Hz, *J* = 6.5 Hz, 1H, HC*H*N), 4.39 (d, *J* = 11.1 Hz, 1H, *H*CH-Ph), 4.24 (d, *J* = 11.1 Hz, 1H, HC*H*-Ph), 4.17–4.10 (m, 1H, PCC*H*), 4.03 (dd, *J* = 9.0 Hz, *J* = 5.5 Hz, 1H, PC*H*), 1.80–1.00 (very br s, 3H, NH_2_ and OH); ^13^C-NMR (151 MHz, CDCl_3_): δ = 159.16, 153.40, 151.42, 142.23, 141.60, 136.66, 135.80 (d, *J* = 5.8 Hz), 135.70 (d, *J* = 5.8 Hz), 128.71, 128.68, 128.43, 128.17, 128.14, 128.01, 125.02, 124.68, 77.64 (d, *J* = 5.2 Hz), 72.73, 68.64 (d, *J* = 7.4 Hz, PO*C*), 68.45 (d, *J* = 7.4 Hz, PO*C*), 67.77 (d, *J* = 161.6 Hz, PC), 50.28 (d, *J* = 5.8 Hz, PCC*C*), 38.56; ^31^P-NMR (121.5 MHz, CDCl_3_): δ = 22.14 ppm. Anal. calcd. for C_32_H_32_ClN_8_O_5_P: C, 56.93; H, 4.78; N, 16.60. Found: C, 56.71; H, 4.71; N, 16.31.

### 3.3. General Procedure for Transformation ***17*** into ***16***

The respective 2-amino-6-chloropurine derivative **17a**‒**17j** (1.00 mmol) was dissolved in a 75% aqueous solution of trifluoroacetic acid (6 mL) and left at room temperature overnight. The solvent was removed, and the residue was co-evaporated with water and subsequently with ethanol to give pure guanine derivatives **16a**‒**16j**.

*Diethyl {4-[(2-amino-6-oxo-1,6-dihydro-9H-purin-9-yl)methyl]-1H-1,2,3-triazol-1-yl}methylphosphonate* (**16a**): A white powder, yield 98%; m.p. = 135–138 °C; IR (KBr): ν = 3330, 3131, 2988, 2935, 1720, 1639, 1606, 1021 cm^−1^; ^1^H-NMR (300 MHz, CD_3_OD): δ = 8.03 (s, 1H), 7.80 (s, 1H, HC5′), 5.37 (s, 2H, CH_2_), 5.02 (d, *J* = 13.1 Hz, 2H, PCH_2_), 4.16–4.07 (m, 4H, 2 × POC*H*_2_CH_3_), 1.25 (t, *J* = 7.1 Hz, 6H, 2 × POCH_2_C*H*_3_); ^13^C-NMR (151 MHz, CD_3_OD): δ = 155.52, 154.83, 150.53, 141.37, 137.21, 125.53, 109.68, 63.64 (d, *J* = 6.6 Hz, POC), 45.08 (d, *J* = 155.0 Hz, PC), 37.72, 15.28 (d, *J* = 5.5 Hz, POC*C*); ^31^P-NMR (121.5 MHz, CD_3_OD): δ = 18.13 ppm. Anal. calcd. for C_13_H_19_N_8_O_4_P: C, 40.84; H, 5.01; N, 29.31. Found: C, 40.98; H, 4.90; N, 29.44.

*Diethyl 2-{4-[(2-amino-6-oxo-1,6-dihydro-9H-purin-9-yl)methyl]-1H-1,2,3-triazol-1-yl}ethylphosphonate* (**16b**): A thick resin, yield 92%; IR (film): ν = 3441, 3129, 2986, 1692, 1537, 1480, 1377, 1204, 1051, 1224 cm^−1^; ^1^H-NMR (300 MHz, CD_3_OD): δ = 8.72 (s, 1H), 8.17 (s, 1H, HC5′), 5.48 (s, 2H, CH_2_), 4.66 (dt, *J* = 13.4 Hz, *J* = 7.4 Hz, 2H, PCH_2_C*H*_2_), 4.09–3.99 (m, 4H, 2 × POC*H*_2_CH_3_), 2.52 (dt, *J* = 18.3 Hz, *J* = 7.4 Hz, 2H, PC*H*_2_), 1.25 (t, *J* = 7.0 Hz, 6H, 2 × POCH_2_C*H*_3_); ^13^C-NMR (151 MHz, CD_3_OD): δ = 160.27, 155.40, 150.63, 141.18, 137.39, 124.70, 110.47, 62.34 (d, *J* = 6.6 Hz, POC), 44.29 (d, *J* = 2.9 Hz, PC*C*), 38.99, 25.70 (d, *J* = 142.0 Hz, PC), 15.20 (d, *J* = 5.8 Hz, POC*C*); ^31^P-NMR (121.5 MHz, CD_3_OD): δ = 28.14 ppm. Anal. calcd. for C_14_H_21_N_8_O_4_P: C, 42.43; H, 5.34; N, 28.27. Found: C, 42.60; H, 5.55; N, 28.40.

*Diethyl 3-{4-[(2-amino-6-oxo-1,6-dihydro-9H-purin-9-yl)methyl]-1H-1,2,3-triazol-1-yl}propylphosphonate* (**16c**): Thick resin, yield 92%; IR (film): ν = 3437, 3134, 2940, 1693, 1538, 1479, 1376, 1228, 1053, 1025 cm^−1^; ^1^H-NMR (300 MHz, CD_3_OD): δ = 8.03 (s, 1H, HC5′), 7.89 (s, 1H), 5.36 (s, 2H, CH_2_), 4.47 (t, *J* = 6.9 Hz, 2H, PCH_2_CH_2_C*H*_2_), 4.15–4.00 (m, 4H, 2 × POC*H*_2_CH_3_), 2.22–2.08 (m, 2H, PCH_2_C*H*_2_), 1.83–1.71 (m, 2H, PC*H*_2_), 1.28 (t, *J* = 7.0 Hz, 6H, POCH_2_C*H*_3_); ^13^C-NMR (75.5 MHz, CD_3_OD): δ = 155.42, 155.07, 150.65, 141.14, 137.25, 124.56, 110.09, 62.09 (d, *J* = 6.7 Hz, POC), 53.50, 49.96 (d, *J* = 18.2 Hz, PCC*C*), 39.15, 23.20 (d, *J* = 4.4 Hz, PC*C*), 21.57 (d, *J* = 143.5 Hz, PC), 15.34 (d, *J* = 5.7 Hz, POC*C*); ^31^P-NMR (121.5 MHz, CD_3_OD): δ = 32.57 ppm. Anal. calcd. for C_15_H_23_N_8_O_4_P: C, 43.90; H, 5.65; N, 27.31. Found: C, 44.12; H, 5.77; N, 27.48.

*Diethyl 4-{4-[(2-amino-6-oxo-1,6-dihydro-9H-purin-9-yl)methyl]-1H-1,2,3-triazol-1-yl}butylphosphonate* (**16d**): An yellowish powder, yield 94%; m.p. = 106–108 °C; IR (KBr): ν = 3330, 3200, 3115, 2936, 2739, 1731, 1694, 1634, 1483, 1388, 1221, 1025 cm^−1^; ^1^H-NMR (300 MHz, CD_3_OD): δ = 8.03 (s, 1H, HC5′), 7.95 (s, 1H), 5.37 (s, 2H, CH_2_), 4.42 (t, *J* = 6.9 Hz, 2H, PCCCC*H*_2_), 4.13–3.96 (m, 4H, 2 × POC*H*_2_CH_3_), 2.04 (qu, *J* = 6.9 Hz, 2H, PCCC*H*_2_), 1.80–1.73 (m, 2H, PC*H*_2_), 1.64–1.42 (m, 2H, PCC*H*_2_), 1.24 (t, *J* = 7.1 Hz, 6H, 2 × POCH_2_C*H*_3_); ^13^C-NMR (151 MHz, CD_3_OD): δ = 155.77, 154.21, 150.35, 140.65, 137.06, 124.61, 108.53, 61.92 (d, *J* = 6.6 Hz, 2 × POC), 49.53, 39.48, 30.20 (d, *J* = 15.8 Hz, PCC*C*), 23.68 (d, *J* = 140.9 Hz, PC), 19.07 (d, *J* = 5.2 Hz, PC*C*), 15.37 (d, *J* = 5.7 Hz, POC*C*); ^31^P-NMR (121.5 MHz, CD_3_OD): δ = 32.62 ppm. Anal. calcd. for C_16_H_25_N_8_O_4_P: C, 45.28; H, 5.94; N, 26.40. Found: C, 45.40; H, 6.07; N, 26.55.

*Diethyl 2-{4-[(2-amino-6-oxo-1,6-dihydro-9H-purin-9-yl)methyl]-1H-1,2,3-triazol-1-yl}-1-hydroxyethylphosphonate* (**16e**): White powder, yield 98%; m.p. > 265 °C; IR (KBr): ν = 3428, 3134, 3063, 2988, 1695, 1613, 1539, 1220, 1047, 1019 cm^−1^; ^1^H-NMR (300 MHz, CD_3_OD): δ = 9.03 (s, 1H), 8.24 (s, 1H), 5.03 (s, 2H, CH_2_), 4.77 (ddd, *J* = 14.5 Hz, *J* = 7.1 Hz, *J* = 3.5 Hz, 1H, PCC*H*_a_H_b_), 4.60 (ddd, *J* = 14.5 Hz, *J* = 9.5 Hz, *J* = 6.6 Hz, 1H, PCCH_a_*H*_b_), 4.37 (dt, *J* = 9.5 Hz, *J* = 3.5 Hz, 1H, PCH), 4.25–4.14 (m, 4H, 2 × POC*H*_2_CH_3_), 1.33 (t, *J* = 7.1 Hz, 6H, 2 × POCH_2_C*H*_3_); ^13^C-NMR (151 MHz, CD_3_OD): δ = 155.67, 154.47, 150.54, 140.52, 137.10, 125.84, 108.92, 66.72 (d, *J* = 167.5 Hz, PC), 63.49 (d, *J* = 6.9 Hz, POC), 63.28 (d, *J* = 6.9 Hz, POC), 51.65 (d, *J* = 9.9 Hz, PC*C*), 39.27, 15.38 (d, *J* = 4.9 Hz, 2 × POC*C*); ^31^P-NMR (121.5 MHz, CD_3_OD): δ = 21.83 ppm. Anal. calcd. for C_14_H_21_N_8_O_5_P × H_2_O: C, 39.07; H, 5.39; N, 26.04. Found: C, 39.19; H, 5.30; N, 26.18.

*Diethyl 3-{4-[(2-amino-6-oxo-1,6-dihydro-9H-purin-9-yl)methyl]-1H-1,2,3-triazol-1-yl}-2-hydroxypropylphosphonate* (**16f**): White powder, yield 98%; m.p. = 144–147 °C; IR (KBr): ν = 3346, 3218, 3218, 2985, 1618, 1564, 1468, 1409, 1220, 1025 cm^−1^; ^1^H-NMR (300 MHz, CD_3_OD): δ = 8.71 (s, 1H), 8.13 (s, 1H), 5.49 (s, 2H), 4.60 (dd, *J* = 13.7 Hz, *J* = 3.2 Hz, 1H, PCCC*H*_a_H_b_), 4.42 (dd, *J* = 13.7 Hz, *J* = 7.6 Hz, 1H, PCCCH_a_*H*_b_), 4.38–4.27 (m, 1H, PCCH), 4.18–4.06 (m, 4H, 2 × POC*H*_2_CH_3_), 2.15 (ddd, *J* = 18.8 Hz, *J* = 15.5 Hz, *J* = 5.5 Hz, 1H, PC*H*_a_H_b_), 2.02 (ddd, *J* = 18.8 Hz, *J* = 15.5 Hz, *J* = 7.2 Hz, 1H, PCH_a_*H*_b_), 1.33 (t, *J* = 7.1 Hz, 3H, POCH_2_C*H*_3_), 1.32 (t, *J* = 7.1 Hz, 3H, POCH_2_C*H*_3_); ^13^C-NMR (151 MHz, CD_3_OD): δ = 155.35, 155.30, 151.24, 140.86, 137.26, 125.52, 112.00, 65.30 (d, *J* = 2.5 Hz, PC*C*), 62.06 (d, *J* = 6.4 Hz, POC), 62.24 (d, *J* = 6.4 Hz, POC), 55.95 (d, *J* = 14.3 Hz, PCC*C*), 39.00, 30.51 (d, *J* = 140.6 Hz, PC), 15.28 (d, *J* = 6.1 Hz, 2 × POC*C*); ^31^P-NMR (121.5 MHz, CD_3_OD): δ = 29.71 ppm. Anal. calcd. for C_15_H_23_N_8_O_5_P × 2H_2_O: C, 38.96; H, 5.89; N, 24.23. Found: C, 39.16; H, 5.84; N, 24.33.

*Diethyl 3-{4-[(2-amino-6-oxo-1,6-dihydro-9H-purin-9-yl)methyl]-1H-1,2,3-triazol-1-yl}-1-hydroxypropylphosphonate* (**16g**): White powder, yield 98%; m.p. = 150–153 °C; IR (KBr): ν = 3330, 3208, 3133, 2989, 2932, 1728, 1690, 1643, 1572, 1410, 1204, 1023 cm^−1^; ^1^H-NMR (300 MHz, CD_3_OD): δ = 8.44 (s, 1H), 8.10 (s, 1H), 5.44 (s, 2H, CH_2_), 4.60 (dd, *J* = 7.9 Hz, *J* = 5.9 Hz, 2H, PCCC*H*_2_), 4.21–4.08 (m, 4H, 2 × POC*H*_2_CH_3_), 3.80 (ddd, *J* = 10.5 Hz, *J* = 7.1 Hz, *J* = 3.2 Hz, 1H, PC*H*), 2.43–2.03 (m, 2H, PCCH_2_), 1.31 (t, *J* = 7.1 Hz, 3H, POCH_2_C*H*_3_), 1.30 (t, *J* = 7.1 Hz, 3H, POCH_2_C*H*_3_); ^13^C-NMR (151 MHz, CD_3_OD): δ = 155.95, 154.99, 151.28, 141.57, 137.50, 124.47, 113.12, 63.67 (d, *J* = 168.1 Hz, PC), 63.09 (d, *J* = 7.1 Hz, POC), 62.73 (d, *J* = 7.1 Hz, POC), 46.42 (d, *J* = 15.6 Hz, PCC*C*), 38.76, 31.79 (d, *J* = 4.1 Hz, PC*C*) 15.43 (d, *J* = 5.4 Hz, POC*C*), 15.39 (d, *J* = 5.4 Hz, POC*C*); ^31^P-NMR (121.5 MHz, CD_3_OD): δ = 25.35 ppm. Anal. calcd. for C_15_H_23_N_8_O_5_P × 2H_2_O: C, 38.96; H, 5.89; N, 24.23. Found: C, 38.72; H, 5.91; N, 24.39.

*Diethyl 2-{4-[(2-amino-6-oxo-1,6-dihydro-9H-purin-9-yl)methyl]-1H-1,2,3-triazol-1-yl}ethoxymethyphosphonate* (**16h**): White powder, yield 98%; m.p. = 89–92 °C; IR (KBr): ν = 3330, 3136, 2992, 2934, 1727, 1649, 1601, 1206, 1023 cm^−1^; ^1^H-NMR (300 MHz, CD_3_OD): δ = 8.91 (s, 1H), 8.20 (s, 1H), 5.52 (s, 2H, CH_2_), 4.63 (t, *J* = 4.7 Hz, 2H, PCH_2_OCH_2_C*H*_2_), 4.11–4.01 (m, 4H, 2 × POC*H*_2_CH_3_), 3.99 (t, *J* = 4.7 Hz, 2H, PCOC*H*_2_CH_2_), 3.87 (d, *J* = 8.5 Hz, 2H, PCH_2_O), 1.25 (t, *J* = 7.1 Hz, 6H, 2 × POCH_2_C*H*_3_); ^13^C-NMR (151 MHz, CD_3_OD): δ = 155.52, 154.96, 150.52, 140.89, 137.19, 125.06, 109.71, 70.94 (d, *J* = 6.6 Hz, PO*C*), 70.86 (d, *J* = 6.6 Hz, PO*C*), 64.06 (d, *J* = 166.6 Hz, PC), 62.81 (d, *J* = 6.6 Hz, PCO*C*), 49.94, 39.15, 15.35 (d, *J* = 5.5 Hz, POC*C*); ^31^P-NMR (121.5 MHz, CD_3_OD): δ = 22.51 ppm. Anal. calcd. for C_15_H_23_N_8_O_5_P×H_2_O: C, 40.54; H, 5.67; N, 25.22. Found: C, 40.44; H, 5.50; N, 25.53.

*Diethyl 2-{4-[(2-amino-6-oxo-1,6-dihydro-9H-purin-9-yl)methyl]-1H-1,2,3-triazol-1-yl}ethoxyethyphosphonate* (**16i**): Colorless oil, yield 98%; IR (film): ν = 3318, 3133, 2987, 2932, 1706, 1639, 1604, 1202, 1051, 1027 cm^−1^; ^1^H-NMR (300 MHz, CD_3_OD): δ = 8.96 (s, 1H), 8.22 (s, 1H), 5.02 (s, 2H, CH_2_), 4.59 (t, *J* = 5.3 Hz, 2H, PCH_2_CH_2_OCH_2_C*H*_2_), 4.08–3.98 (m, 4H, 2 × POC*H*_2_CH_3_), 3.86 (t, *J* = 5.3 Hz, 2H, PCCOC*H*_2_CH_2_), 3.68 (dt, *J* = 16.4 Hz, *J* = 6.8 Hz, 2H, PCH_2_C*H*_2_O), 2.10 (dt, *J* = 18.2 Hz, *J* = 6.8 Hz, 2H, PC*H*_2_CH_2_O), 1.27 (t, *J* = 7.1 Hz, 6H, 2 × POCH_2_C*H*_3_); ^13^C-NMR (151 MHz, CD_3_OD): δ = 155.39, 154.20, 150.32, 141.04, 137.41, 125.01, 113.80, 68.51, 64.47 (d, *J* = 3.0 Hz, PC*C*O), 61.96 (d, *J* = 6.5 Hz, PO*C*), 50.10, 39.09, 25.70 (d, *J* = 140.3 Hz, PC), 15.29 (d, *J* = 6.4 Hz, POC*C*); ^31^P-NMR (121.5 MHz, CD_3_OD): δ = 30.92 ppm. Anal. calcd. for C_16_H_25_N_8_O_5_P: C, 43.64; H, 5.72; N, 25.44. Found: C, 43.74; H, 5.86; N, 25.66.

*Diethyl 2-{4-[(2-amino-6-oxo-1,6-dihydro-9H-purin-9-yl)methyl]-1H-1,2,3-triazol-1-yl}acetamidomethyphosphonate* (**16j**): White powder, yield 98%; m.p. > 230 °C; IR (KBr): ν = 3331, 3127, 2990, 2930, 1729, 1680, 1635, 1563, 1478, 1389, 1209, 1024 cm^−1^; ^1^H-NMR (300 MHz, CD_3_OD): δ = 8.84 (s, 1H), 8.17 (s, 1H), 5.52 (s, 2H, CH_2_), 5.23 (s, 2H), 4.18–4.08 (m, 4H, 2 × POC*H*_2_CH_3_), 3.74 (brd, *J* = 11.9 Hz, 2H, PC*H*_2_NH), 1.30 (t, *J* = 6.9 Hz, 6H, 2 × POCH_2_C*H*_3_); ^13^C-NMR (151 MHz, CD_3_OD): δ = 166.37 (d, *J* = 3.4 Hz, C=O), 155.70, 154.66, 149.94, 140.70, 137.04, 126.11, 109.88, 62.84 (d, *J* = 6.5 Hz, PO*C*), 51.59, 39.25, 34.37 (d, *J* = 158.5 Hz, PC), 15.30 (d, *J* = 5.7 Hz, POC*C*); ^31^P-NMR (121.5 MHz, CD_3_OD): δ = 22.20 ppm. Anal. calcd. for C_15_H_22_N_9_O_5_P: C, 41.00; H, 5.05; N, 28.69. Found: C, 40.66; H, 4.89; N, 28.50.

### 3.4. Antiviral Activity Assays

The compounds were evaluated against the following viruses: herpes simplex virus type 1 (HSV-1) strain KOS, thymidine kinase-deficient (TK^−^) HSV-1 KOS strain resistant to acyclovir (ACV^r^), herpes simplex virus type 2 (HSV-2) strains Lyons and G, varicella-zoster virus (VZV) strain Oka, TK^−^ VZV strain 07−1, human cytomegalovirus (HCMV) strains AD-169 and Davis, vaccinia virus Lederle strain, respiratory syncytial virus (RSV) strain Long, vesicular stomatitis virus (VSV), Coxsackie B4, para-influenza 3, influenza virus A (subtypes H1N1, H3N2), influenza virus B, reovirus-1, Sindbis, reovirus-1, Punta Toro, human immunodeficiency virus type 1 strain III_B_ and human immunodeficiency virus type 2 strain ROD. The antiviral, other than anti-HIV, assays were based on the inhibition of the virus-induced cytopathicity or plaque formation in human embryonic lung (HEL) fibroblasts, African green monkey cells (Vero), human epithelial cells (HeLa) or Madin-Darby canine kidney cells (MDCK). Confluent cell cultures in microtiter 96-well plates were inoculated with 100 CCID_50_ of virus (1 CCID_5__0_ being the virus dose to infect 50% of the cell cultures) or with 20 plaque forming units (PFU) (VZV) in the presence of varying concentrations of the test compounds. Viral cytopathicity or plaque formation was recorded as soon as it reached completion in the control virus-infected cell cultures that were not treated with the test compounds. Antiviral activity was expressed as the EC_50_ or compound concentration required to reduce virus-induced cytopathogenicity or viral plaque formation by 50%.

### 3.5. Cytostatic Activity Assays

All assays were performed in 96-well microtiter plates. To each well were added (5−7.5) × 10^4^ tumor cells and a given amount of the test compound. The cells were allowed to proliferate for 48 h (murine leukemia L1210 cells) or 72 h (human lymphocytic CEM and human cervix carcinoma HeLa cells) at 37 °C in a humidified CO_2_-controlled atmosphere. At the end of the incubation period, the cells were counted in a Coulter counter. The IC_50_ (50% inhibitory concentration) was defined as the concentration of the compound that inhibited cell proliferation by 50%.

## 4. Conclusions 

A novel series of {4-[(2-amino-6-chloro-9*H*-purin-9-yl)methyl]-1*H*-1,2,3-triazol-1-yl}alkylphosphonates **17** was synthesized and subsequently transformed into {4-[(2-amino-6-oxo-1,6-dihydro-9*H*-purin-9-yl)methyl]-1*H*-1,2,3-triazol-1-yl}alkylphosphonates **16** as acyclic analogues of guanosine. Evaluation of the antiviral activity of phosphonates **17a**–**k**, as well as **16a**–**j** was performed against a broad variety of DNA and RNA viruses; however, none of them was found active at concentrations up to 250 μM. The cytostatic properties of Compounds **17a**–**k** and **16a**–**j** were studied on L1210, CEM and HeLa cell lines. Among them, Compounds (1*R*,2*S*)-**17k** and (1*S*,2*S*)-**17k** were moderately active toward L1210 and CEM cells (IC_50_ in the 16–30 µM range).
